# Isolation and Bioactivity Analysis of Ethyl Acetate Extract from *Acer tegmentosum* Using In Vitro Assay and On-Line Screening HPLC-ABTS^+^ System

**DOI:** 10.1155/2014/150509

**Published:** 2014-10-15

**Authors:** Kwang Jin Lee, Na-Young Song, You Chang Oh, Won-Kyung Cho, Jin Yeul Ma

**Affiliations:** Korean Institute of Oriental Medicine (KIOM), KM-Based Herbal Drug Development Group, 1672 Yuseongdae-ro, Yuseong-gu, Daejeon 305-811, Republic of Korea

## Abstract

The *Acer tegmentosum* (3 kg) was extracted using hot water, and the freeze-dried extract powder was partitioned successively using dichloromethane (DCM), ethyl acetate (EA), butyl alcohol (*n*-BuOH), and water. From the EA extract fraction (1.24 g), five phenolic compounds were isolated by the silica gel, octadecyl silica gel, and Sephadex LH-20 column chromatography. Based on spectroscopic methods such as ^1^H-NMR, ^13^C-NMR, and LC/MS the chemical structures of the compounds were confirmed as feniculin (**1**), avicularin (**2**), (+)-catechin (**3**), (−)-epicatechin (**4**), and 6′-*O*-galloyl salidroside (**5**). Moreover, a rapid on-line screening HPLC-ABTS^+^ system for individual bioactivity of the EA-soluble fraction (five phenolic compounds) was developed. The results indicated that compounds **1** and **2** were first isolated from the *A. tegmentosum*. The anti-inflammatory activities and on-line screening HPLC-ABTS^+^ assay method of these compounds in LPS-stimulated murine macrophages were rapid and efficient for the investigation of bioactivity of *A. tegmentosum*.

## 1. Introduction

Traditional remedies based on natural products could be traced back over five millennia to written documents of the early civilizations [1]. Particularly, traditional Korean and Chinese oriental medicines herbs (OMHs) have attracted interest and acceptance in many countries with the merits of a few side effects, affordability, and local availability. Moreover, their long historical clinical practice and reliable therapeutic efficacy make them excellent sources for discovering natural bioactive compounds [2]. Among them,* Acer *(*A.*)* tegmentosum* (Aceraceae, Sancheong-mok in Korean) is a type of deciduous tree distributed in the Northeast Asia including Korea, Russia, and China [3]. In Korea, the leaves and stem of* A. tegmentosum *have been traditionally used for the treatment of hepatic disorders such as hepatitis, hepatic cancer, hepatic cirrhosis, and liver detoxification [4]. Previous studies have shown that extracts of the stem of* A. tegmentosum *possess various pharmacological properties such as antioxidative, anti-inflammatory, antigastrophatic, antiadipogenic, anticancer, and cytotoxic activities [5–7]. However, the phytochemical constituents and efficient method for investigating bioactivity of* A. tegmentosum *have not been reported.

Also, a variety of approaches have been developed for the extraction of useful components from* A. tegmentosum*, for instance, soxhlet extraction (SE), heating reflux extraction (HEE), supercritical fluid extraction (SFE), ultrasonic assisted extraction (UAE), and microwave-assisted extraction (MAE) [8–10]. Water, methanol, ethanol, and ethyl acetate (EA) were commonly used solvents for the extraction of bioactive compounds from plant materials and OMHs. The identification and relative amounts of five types of compounds in* A. tegmentosum *were determined by LC-MS, NMR, and on-line screening HPLC-ABTS^+^ assay [1]. These techniques included on-line screening with HPLC post-column assay involving the ABTS^+^ radical technique [11], allowing spectrophotometric monitoring of bioactive compounds. Generally, DPPH (ABTS) radical is another simple, rapid on-line method for the detection of antioxidants from crude plant extracts [12]. It combines HPLC with an assay involving a stable radical species [1,1-diphenyl-2-picrylhydrazyl radical (DPPH) and 2,2′-azinobis-(3-ethylbenzothiazoline-6-sulfonic acid) radical cation (ABTS)] in the HPLC-DPPH (ABTS) method [13, 14]. Moreover, this method was successfully applied for the screening and identification of natural bioactive compounds from complex mixtures, particularly for the extracts of OMHs [15–17]. The chemical structures of the five types of compounds were confirmed by spectroscopic methods such as ^1^H-NMR, ^13^C-NMR, and LC/MS [18]. In this study, five phenolic compounds were isolated from hot water extract of* A. tegmentosum* bychromatographic separation. Using spectroscopic methods, the structures of these compounds were determined as feniculin (**1**), avicularin (**2**), (+)-catechin (**3**), (−)-epicatechin (**4**), and 6′-*O*-galloyl salidroside (**5**). Moreover, we investigated their anti-inflammatory effects on LPS-stimulated RAW 264.7 cells. We also investigated the applications of on-line screening HPLC-ABTS^+^ assays for bioactivity screening to find a more practical approach toward the use of on-line screening HPLC-ABTS^+^ assays for the rapid pinpointing of peaks in chromatograms corresponding to bioactive compounds.

## 2. Experimental

### 2.1. Reagents and Materials

**Figure 1:**
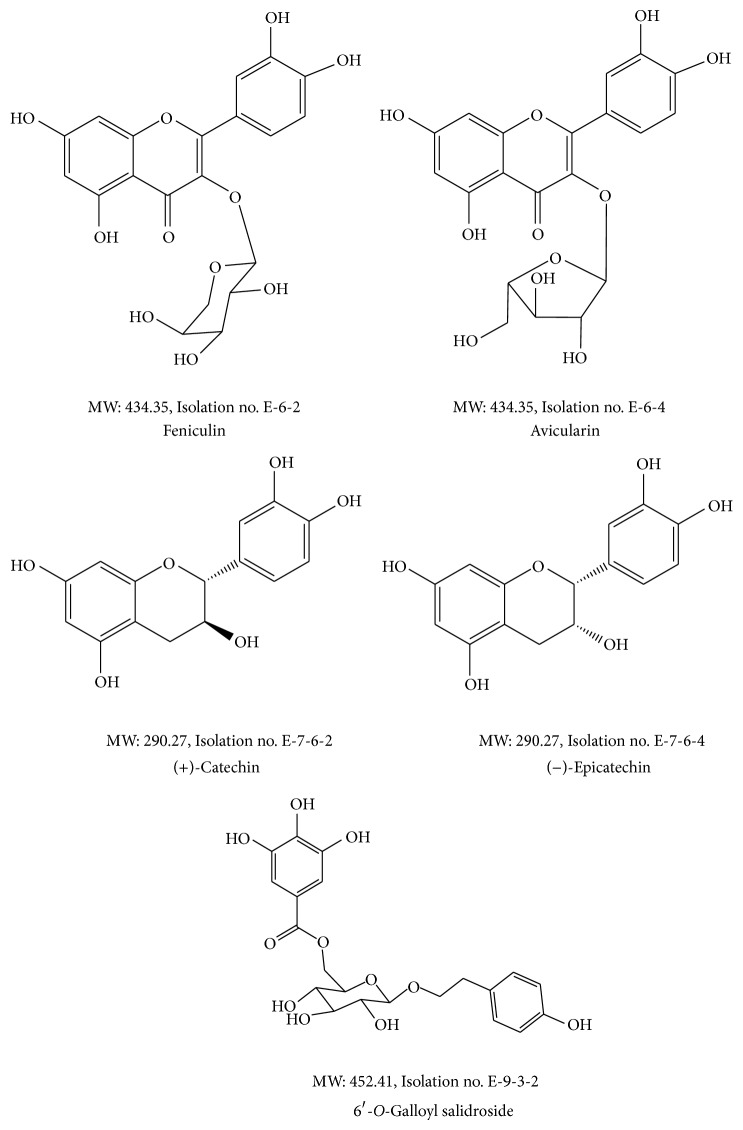
Chemical structure of five types of compounds from* Acer tegmentosum*.

The stem of* A. tegmentosum *was purchased from Yeongcheon traditional herbal market (Gyeongsangbuk-do, Yeongcheon, Korea). All voucher specimens were deposited in the herbal bank of KM-Based Herbal Drug Development Group, Korea Institute of Oriental Medicine. The following reagents were used for radical scavenging assays: ABTS^+^ (2,2′-azino-bis-3-ethylbenzothiazoline-6-sulfonic acid), potassium persulfate, and trifluoroacetic acid (TFA) were purchased from Sigma Co. (USA). The HPLC grade methanol (MeOH) and acetonitrile (ACN) were purchased from J. T. Baker (Philipsburg, NJ, USA). The hexane, dichloromethane (DCM), ethyl acetate (EA), and normal butyl alcohol (*n*-BuOH) were purchased from Daejung chemical (Gyeonggi-do, Shiheung, Korea). The triple distilled water was filtered with a 0.2 *μ*m membrane filter (Advantec, Tokyo, Japan) before analysis. Materials for cell culture were obtained from Lonza (Basel, Switzerland). LPS, Bovine serum albumin (BSA), and 3-(4,5-dimethylthiazol-2-yl)-2,5-diphenyltetrazolium bromide (MTT) were purchased from Sigma (St. Louis, MO, USA). Antibodies for ELISA were obtained from eBioscience (San Diego, CA, USA). The chemical structures of five types of compounds are shown in Figure 1.

### 2.2. Standard Sample Preparation

The high purity isolated compounds (higher than > 95%) were prepared by dissolving 2 mg of the standard chemicals feniculin (**1**), avicularin (**2**), (+)-catechin (**3**), (−)-epicatechin (**4**), and 6′-*O*-galloyl salidroside (**5**) in 10 mL of methanol and adjusting the concentration to 200 ppm.

### 2.3. ABTS^+^ Sample Preparation

A 2 mM ABTS stock solution containing 3.5 mM potassium persulfate was prepared and was kept in the dark at room temperature for 16 h to allow the completion of radical generation and was then diluted with water (1 : 29, v/v).

### 2.4. Solvent Extraction and Purification

**Figure 2:**
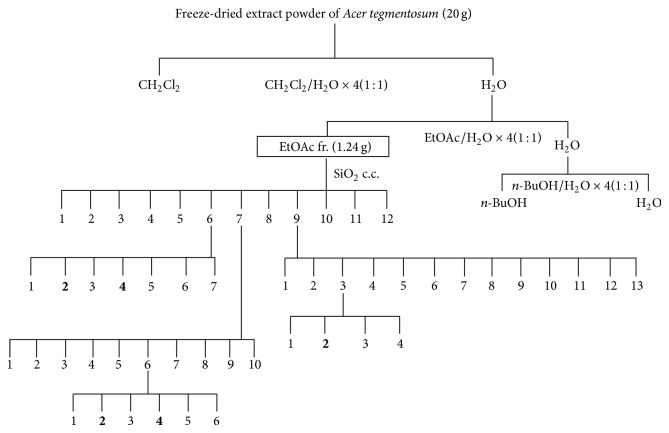
Process of isolation five kind compounds of ethyl acetate extract from* Acer tegmentosum*.

Dry samples (3 Kg) from the powders of the* A. tegmentosum* were loaded (10-times volume) in hot water extraction system. The extraction was performed by heating for 3 h at 100°C (Gyeongseo Extractor Cosmos-600, Inchon, Korea). Then, the solution was filtered using standard testing sieves (150 *μ*m, Retsch, Haan, Germany), freeze-dried, and maintained in desiccators at 4°C prior to use. For large amount of extractions, 20 g freeze-dry samples were loaded (1 : 1, extracted thrice) and extracted successively using DCM, EA, and* n*-BuOH. The contents of feniculin (**1**), avicularin (**2**), (+)-catechin (**3**), (−)-epicatechin (**4**), and 6′-*O*-galloyl salidroside (**5**) in* A. tegmentosum *were remarkably higher in the EA extract (1.24 g). Then, the samples were filtered through a 0.2 *μ*m membrane filter prior to on-line screening HPLC-ABTS^+^ analysis. The extraction and purification processes from* A. tegmentosum *are shown in Figure 2.

### 2.5. Analysis System

NMR spectra were obtained using a Varian Inova 400 MHz and 600 MHz NMR (Varian, USA). The isolated compounds feniculin (**1**), avicularin (**2**), (+)-catechin (**3**), (−)-epicatechin (**4**), and 6′-*O*-galloyl salidroside (**5**) were confirmed viaLC-MS analysis using Agilent 1100 + G1958 (Agilent, USA) LC systems (Table 1). Open column chromatography was performed using silica gel (Kieselgel 60, Merck, Germany) and octadecylsilane (ODS) Li-Chropre RP-18 (Merck, Germany). Molecular sieve column chromatography was performed using Sephadex LH-20 (Fluka, USA). Thin layer chromatography (TLC) analysis was performed using silica gel glass plates (Kieselgel 60 F_254_ and RP-18 F_254S_, Merck, Germany) and developed using a mobile phase composed of chloroform-methanol-water and stained using 10% H_2_SO_4_ to detect the EA extract and pure compounds.

### 2.6. On-Line Screening HPLC-ABTS^+^ Assay Analysis

**Figure 3:**
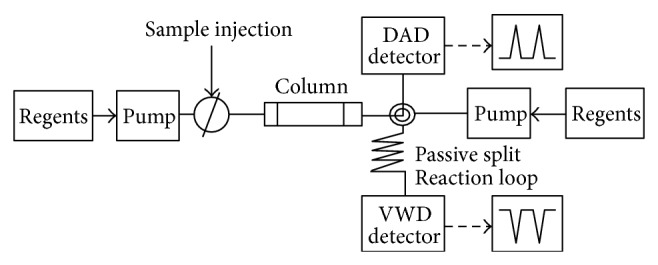
Schematic of on-line screening HPLC-ABTS^+^ system.

*A. tegmentosum *extract was injected into a Dionex Ultimate 3000 HPLC system (Thermo Scientific). The chromatographic columns used in this experiment were commercially available and were purchased from RS-tech (0.46 × 25 cm, 5 *μ*m, C_18_, Daejeon, Korea). The injection volume was 10 *μ*L, and the flow rate of the mobile phase was 1.0 mL/min. The wavelength of the UV detector was fixed at 210, 254, 280, and 320 nm. The composition of the mobile phases was A: 99.9% water/trifluoroacetic acid (99.9/0.1 vol%) and B: 100% acetonitrile. The run time was 60 min, and the solvent program was the linear gradient method (90 : 10–60 : 40 A : B vol%, 70 min: initial condition) (Table 2).  Figure 3 shows a schematic diagram of the on-line coupling of a HPLC to a DAD (diode array detector) and the continuous flow ABTS^+^ assay. Then, on-line HPLC was connected to a “T” piece where ABTS^+^ was added. The ABTS^+^ at a flow rate of 0.5 mL/min was delivered using a Dionex ultimate 3000 pump. After mixing through a 1 mL loop, maintained at 40°C, the absorbance was measured using a multiple wavelength detector (MWD) at 734 nm. Data were analyzed using the Chromeleon 7 software.

### 2.7. Cell Culture and Drug Treatment

RAW 264.7 cells were purchased from Korea Cell Line Bank (Seoul, Korea) and grown in RPMI 1640 medium containing 10% FBS and 100 U/mL of antibiotics sulfate. The cells were incubated in humidified 5% CO_2_ atmosphere at 37°C. To stimulate the cells, the medium was changed with fresh RPMI 1640 medium, and LPS (200 ng/mL) was added in the presence or absence of five compounds (10, 30, 50, and 100 *μ*M) for 24 h.

### 2.8. MTT Assay for Cell Viability

Cytotoxicity was analyzed using the MTT assay. Five compounds were added to the cells and incubated for 24 h at 37°C with 5% CO_2_. MTT solutions were added to each well and the cells were incubated for another 4 h. The formazan melted in dimethyl sulfoxide (DMSO), and then the optical density was read at 570 nm using an ELISA reader (Infinite M200, TECAN, Männedorf, Switzerland).

### 2.9. Measurement of NO Production

NO production was analyzed by measuring the nitrite in the supernatants of cultured macrophage cells. The cells were pretreated with five compounds and stimulated with LPS for 24 h. The supernatant was mixed with the same volume of Griess reagent (1% sulfanilamide, 0.1% naphthylethylenediamine dihydrochloride, and 2.5% phosphoric acid) and incubated at room temperature (RT) for 5 min [19]. The absorbance at 570 nm was read.

### 2.10. Determination of TNF-*α*, IL-6, and IL-1*β* Cytokine Production

Cells were seeded at a density of 5 × 10^5^ cells/mL in 24-well culture plates and pretreated with various concentrations of five compounds for 30 min before the LPS stimulation. ELISA plates (Nunc, Roskilde, Denmark) were coated overnight at 4°C with capture antibody diluted in a coating buffer (0.1 M carbonate, pH 9.5) and then washed five times with phosphate-buffered saline (PBS) containing 0.05% Tween 20. The nonspecific protein-binding sites were blocked with assay diluent buffer (PBS containing 10% FBS, pH 7.0) for >1 h. The samples and standards were added to the wells promptly. After 2 hours of incubation at RT or overnight at 4°C, the working detector solution (biotinylated detection antibody and streptavidin-HRP reagent) was added and incubated for 1 h. Subsequently, the substrate solution (tetramethylbenzidine) was added to the wells and incubated for 30 min in the dark before the reaction was quenched with stop solution (NH_3_PO_4_). The optical density was read at 450 nm [19].

### 2.11. Statistical Analysis

The results are expressed as mean ± SE values for the number of experiments. Statistical significance of each treated group was compared to the control and determined by Student's *t*-tests. Each experiment was repeated at least thrice to yield comparable results. Values with *P* < 0.01 and <0.001 were considered significant.

## 3. Results and Discussion

### 3.1. High Purity Isolation and Analysis

The stems of* A. tegmentosum *were extracted using boiling water and then partitioned successively using dichloromethane (DCM), ethyl acetate (EA), normal butyl alcohol (*n*-BuOH), and water (H_2_O). The EA extract (1.24 g) was applied to silica gel c.c. (*φ* 4 × 10 cm) and eluted using chloroform- (CHCl_3−_) MeOH–H_2_O [15 : 3 : 1 (1.2 L) → 13 : 3 : 1 (1.3 L) → 10 : 3 : 1 (1.2 L) → 7 : 3 : 1 (1 L) → 4 : 3 : 1 (1 L) → MeOH]. Eluted fractions were monitored by TLC to produce 12 fractions (OS1E-1–12). Fraction OS1E-6 (55.5 mg) was subjected to Sephadex LH-20 c.c. [*φ* 1.5 × 45 cm, 50% MeOH (180 mL) → 70% MeOH (300 mL) → 100% MeOH] yielding seven fractions (OS1E-6-1–7) and ultimately produced compounds** 1** (OS1E-6-2, 6.0 mg) and** 2** (OS1E-6-4, 5.9 mg). Fraction OS1E-7 (260.3 mg) was subjected to Sephadex LH-20 c.c. [*φ* 1.5 × 44 cm, 80% MeOH (400 mL) → 100% MeOH] yielding 10 fractions (OS1E-7-1–10). Fraction OS1E-7-6 (53.1 mg) was subjected to ODS c.c. (*φ* 1.5 × 8 cm) and eluted with MeOH–H_2_O [1 : 6 (900 mL) → MeOH] to afford compounds** 3** (OS1E-7-6-2, 30.7 mg) and** 4** (OS1E-7-6-4, 14.2 mg). Fraction OS1E-9 (237.5 mg) was subjected to ODS c.c. (*φ* 2.5 × 8 cm) and eluted with MeOH–H_2_O [1 : 3 (450 mL) → 1 : 2 (300 mL) → 1 : 1 (300 mL) → MeOH] yielding 13 fractions (OS1E-9-1–13). Fraction OS1E-9-3 (77.8 mg) was subjected to silica gel c.c. (*φ* 1.5 × 10 cm) and eluted with CHCl_3_–MeOH–H_2_O [10 : 3 : 1 (2.2 L) → 7 : 3 : 1 (1.6 L) → 4 : 3 : 1 (1.4 L) → MeOH] to afford compound** 5** (OS1E-9-3-2, 60.3 mg). Based on the spectroscopic methods, such as ^1^H-NMR, ^13^C-NMR, and LC/MS, the chemical structures of the compounds were confirmed as feniculin (**1**), avicularin (**2**), (+)-catechin (**3**), (−)-epicatechin (**4**), and 6′-*O*-galloyl salidroside (**5**) by comparison of spectral data with those of references. Moreover, the on-line screening HPLC-ABTS^+^ assay method was rapid and efficient for the investigation of bioactivity of the isolated compounds from* Acer tegmentosum.* Compounds** 1**,** 2**,** 3**,** 4**, and** 5** were developed on ODS TLC with 10% H_2_SO_4_ and the expected yellow or brown color was observed for the phenolic compounds.

*Compound* ***1*** *(Feniculin)*. Yellow amorphous powder (MeOH); ESI-MS* m/z* 435.1 [M+H]^+^; ^1^H-NMR (400 MHz, CD_3_OD, *δ*_H_) 7.73 (1H, d,* J *= 1.6 Hz, H-2′), 7.55 (1H, dd,* J *= 8.4, 1.6 Hz, H-6′), 6.88 (1H, d,* J *= 8.4 Hz, H-5′), 6.39 (1H, d,* J *= 1.6 Hz, H-8), 6.19 (1H, d,* J *= 1.6 Hz, H-6), 5.16 (1H, d,* J *= 6.0 Hz, H-1′′), 3.89 (1H, dd,* J *= 8.0, 6.0 Hz, H-2′′), 3.82 (1H, H-5′′a), 3.80 (1H, m, H-4′′), 3.64 (1H, dd,* J *= 8.0, 2.8 Hz, H-3′′), 3.44 (1H, dd,* J *= 13.2, 3.2 Hz, H-5′′b); ^13^C-NMR (100 MHz, CD_3_OD, *δ*_c_); see Table 3. The characterization data of compound** 1** were compared to the literature value and combined with quercetin and arabinopyranoside to confirm the structure as feniculin [20].

*Compound* ***2*** *(Avicularin)*. Yellow amorphous powder (MeOH); ESI-MS* m/z* 435.1 [M+H]^+^; ^1^H-NMR (600 MHz, CD_3_OD, *δ*_H_) 7.52 (1H, d,* J *= 1.8 Hz, H-2′), 7.49 (1H, dd,* J *= 8.4, 1.8 Hz, H-6′), 6.90 (1H, d,* J *= 8.4 Hz, H-5′), 6.38 (1H, d,* J *= 1.8 Hz, H-8), 6.20 (1H, d,* J *= 1.8 Hz, H-6), 5.46 (1H, s, H-1′′), 4.32 (1H, d,* J *= 2.4 Hz, H-2′′), 3.89 (1H, dd,* J *= 4.8, 2.4 Hz, H-3′′), 3.861 (1H, m, H-4′′), 3.494 (2H, t,* J *= 4.2, H-5′′); ^13^C-NMR (150 MHz, CD_3_OD, *δ*_c_); see Table 3. ^1^H-NMR spectrum of compound** 2** showed a similar pattern to compound** 1**. The structure was confirmed by ESI-MS* m/z* 435.1 [M+H]^+^ [21]. Compounds** 1** and** 2** were isolated from the plant for the first time.

*Compound* ***3*** *[(+)-Catechin]*. Brown powder (MeOH); ESI-MS* m/z* 291.0 [M+H]^+^; ^1^H-NMR (600 MHz, CD_3_OD, *δ*_H_) 6.83 (1H, d,* J *= 2.4 Hz, H-2′), 6.76 (1H, d,* J *= 8.4 Hz, H-5′), 6.71 (1H, dd,* J *= 8.4, 2.4 Hz, H-6′), 5.92 (1H, d,* J *=2.4 Hz, H-6), 5.85 (1H, d,* J *= 2.4 Hz, H-8), 4.56 (1H, d,* J *= 7.2 Hz, H-2), 3.97 (1H, m, H-3), 2.50 (1H, dd,* J *= 16.2, 8.4 Hz, H-4a), 2.84 (1H, dd,* J *= 16.2, 6.0 Hz, H-4b); ^13^C-NMR (150 MHz, CD_3_OD, *δ*_c_); see Table 3.

*Compound* ***4*** *[(−)-Epicatechin]*. Brown powder (MeOH); ESI-MS* m/z* 291.0 [M+H]^+^; ^1^H-NMR (600 MHz, CD_3_OD, *δ*_H_) 6.96 (1H, d,* J *= 1.8 Hz, H-2′), 6.79 (1H, dd,* J *= 8.4, 1.8 Hz, H-6′), 6.75 (1H, d,* J *= 8.4 Hz, H-5′), 5.93 (1H, d,* J *= 1.8 Hz, H-6), 5.91 (1H, d,* J *= 1.8 Hz, H-8), 4.80 (1H, brs, H-2), 4.16 (1H, m, H-3), 2.86 (1H, dd,* J *= 16.8, 4.8 Hz, H-4a), 2.73 (1H, dd,* J *= 16.8, 3.0 Hz, H-4b); ^13^C-NMR (150 MHz, CD_3_OD, *δ*_c_); see Table 3. Compounds** 3** and** 4** were compared to the literature data for (+)-catechin and (−)-epicatechin for the structure identification [22]. The structure was confirmed by the presence of* m/z* at 291.0 [M+H]^+^ in the ESI-MS positive mode.

*Compound* ***5*** *(6* ′ *-O-Galloyl salidroside)*. Pale yellow oil (MeOH); ESI-MS* m/z* 453.1 [M+H]^+^; ^1^H-NMR (600 MHz, CD_3_OD, *δ*_H_) 7.10 (2H, br s, H-2′′′, H-6′′′), 6.96 (2H, d,* J *= 8.4 Hz, H-2, 6), 6.65 (2H, d,* J *= 8.4 Hz, H-3, H-5), 6.65 (1H, d,* J *= 8.4 Hz, H-5), 4.52 (1H, dd,* J *= 11.4, 2.4 Hz, H-6′′a), 4.45 (1H, dd,* J *= 11.4, 6.0 Hz, H-6′′b), 4.32 (1H, d,* J *= 7.8 Hz, H-1′′), 3.95 (1H, m, H-8a), 3.70 (1H, m, H-8b), 3.55 (1H, m, H-5′′), 3.42 (2H, t,* J *= 7.8 Hz, H-3′′, 4′′), 3.22 (1H, t,* J *= 8.4 Hz, H-2′′), 2.77 (2H, m, H-7); ^13^C-NMR (150 MHz, CD_3_OD, *δ*_c_); see Table 3. ^1^H-NMR data were consistent with the literature values [23].

### 3.2. On-Line Screening HPLC-ABTS^+^ Assay Analysis

**Figure 4:**
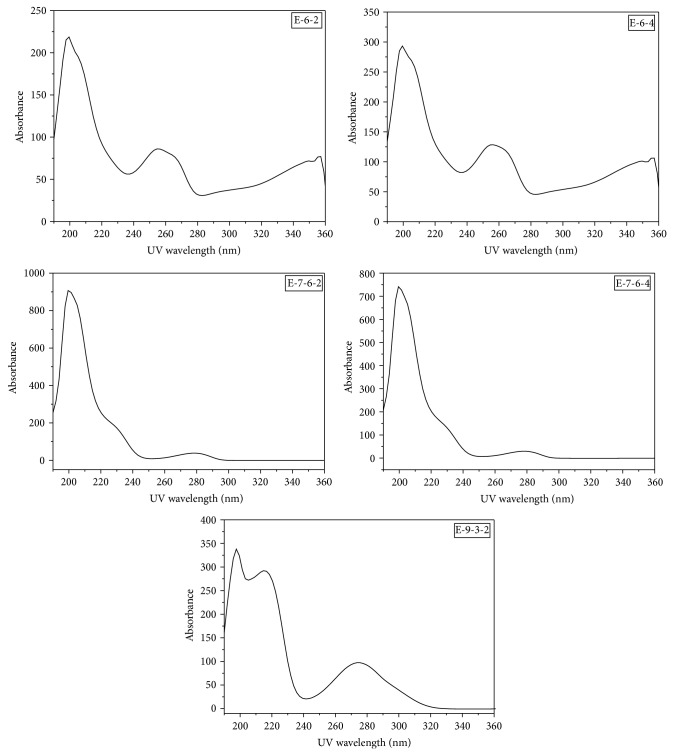
Analysis of UV wavelength from high purity isolation compounds.

**Figure 5:**
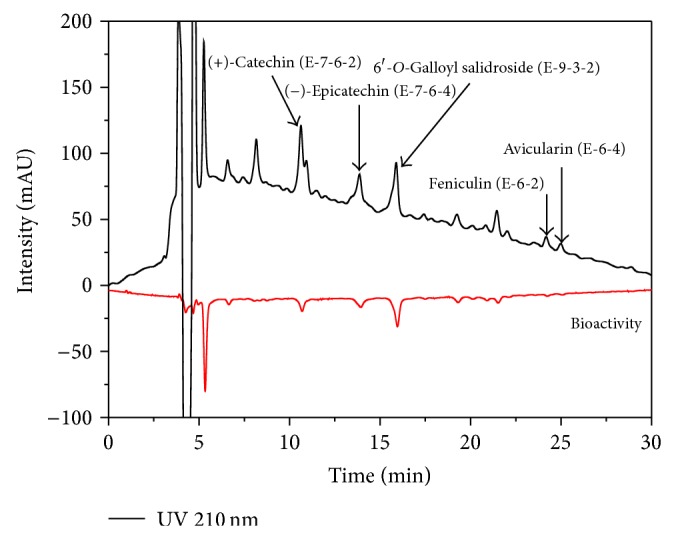
Bioactivity screening of ethyl acetate extract from* Acer tegmentosum* using on-line screening HPLC-ABTS^+^ assay (injection volume: 10 *μ*L; UV wavelength: positive 210 nm; negative 734 nm).

**Figure 6:**
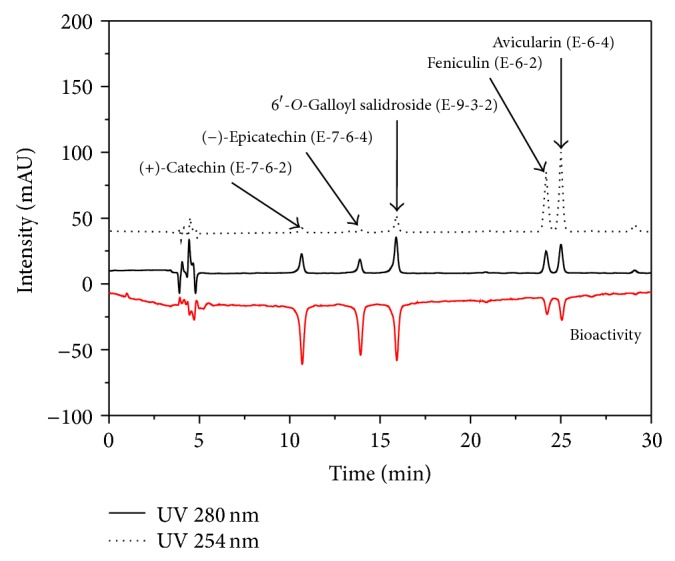
Analysis of high purity compounds of ethyl acetate extract in* Acer tegmentosum* (injection volume: 10 *μ*L; UV wavelength: positive 280 and 254 nm, negative 734 nm; sample concentration 200 ppm).

This study investigated the bioactivity (using ABTS^+^ assay; radical scavenging activity) and anti-inflammatory activity of the five isolated phenolic compounds that were measured. All the compounds** 1**–**5** in the EA fraction (each yield; mg) exhibited antioxidant activities (Table 4). Moreover, this on-line screening HPLC-ABTS^+^ assay method was rapid and efficient for the investigation of bioactivity from* A. tegmentosum* and was obtained from RS-tech (0.46 × 25 cm, 5 *μ*m, C_18_, Daejeon, Korea). The injection volume was 10 *μ*L, and the flow rate of the mobile phase was 1.0 mL/min. The wavelength of the UV detector was fixed at 210, 254, 280, and 320 nm. The five phenolic compounds were characterized by comparing the HPLC UV-DAD maximum absorption peaks of the samples with those of the pure isolation standards (Figure 4). The determination of antioxidant activity from the on-line HPLC–DPPH (ABTS) assay was based on the decrease in absorbance at 517 and 734 nm after the postcolumn reaction of antioxidants separated from HPLC with DPPH (ABTS), and the antioxidants present in a sample would be easily indicated by negative peaks. The composition of the mobile phases was A: 99.9 vol% water/trifluoroacetic acid (99.9/0.1, vol%) and B: 100% acetonitrile. The run time was 60 min, and the solvent program used the linear gradient method (90 : 10–60 : 40 A : B vol%, 70 min: initial condition). The ABTS^+^ flow rate was 0.5 mL/min. The HPLC separated analytes showed a postcolumn reaction with the ABTS^+^ and the reduction was detected as a negative peak using a MWD at 734 nm. The combined UV (positive signals) and ABTS^+^ quenching (negative signals) chromatograms of the different* A. tegmentosum *extracts (200 ppm) are presented in Figures 5 and 6. Several eluted fractions of the five phenolic compounds in the EA extract were detected as feniculin (**1**), avicularin (**2**), (+)-catechin (**3**), (−)-epicatechin (**4**), and 6′-*O*-galloyl salidroside (**5**), giving a positive signal on the UV detector at 210 nm. The other compounds showed hydrogen-donating capacity (negative peak) toward the ABTS^+^ radical at the applied concentration. These results revealed that the method could be applied for quick bioactivity screening, or more precisely, to detect the radical-scavenging activity of compounds, indicating that (+)-catechin (**3**), (−)-epicatechin (**4**), and 6′-*O*-galloyl salidroside (**5**) exhibited bioactivities. 6′-*O*-Galloyl salidroside (**5**) exhibited higher bioactivity, whereas feniculin (**1**) and avicularin (**2**) showed low bioactivity (Figure 5). The retention time (*R*_*t*_) of feniculin (**1**), avicularin (**2**), (+)-catechin (**3**), (−)-epicatechin (**4**), and 6′-*O*-galloyl salidroside (**5**) was 10.700, 13.884, 15.928, 24.217, and 25.062 min, respectively. The bioactivity appeared to be approximately proportional to the concentration of the five phenolic compounds in the extracts. The HPLC analysis conditions for the best separation of the five compounds were successfully established by varying the open column treatment and solvent purification step (Figure 6). This study confirms the feasibility of assessing the bioactivity of specific phytochemicals using an on-line screening HPLC-ABTS^+^ assay method (Table 5). This proposed method was successfully applied for the screening and identification of natural bioactive compounds from* A. tegmentosum.*

### 3.3. LC-MS Analysis Condition

**Figure 7:**
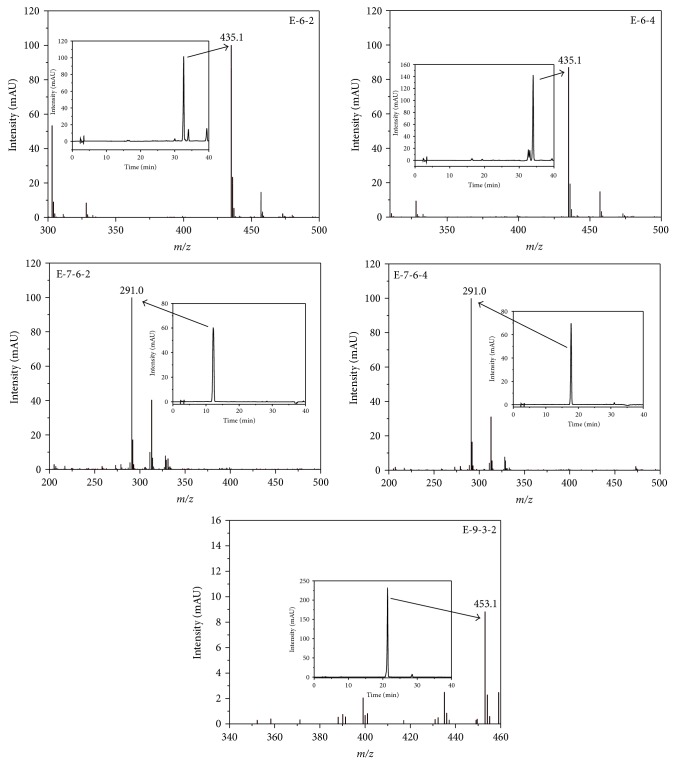
HPLC profile and LC-MS spectra of isolation five kind compounds from* Acer tegmentosum.*

Figure 7 shows the HPLC profile and LC-MS spectra of five isolated phenolic compounds from* A. tegmentosum.* LC-MS analysis indicated that the compounds from* A. tegmentosum *were isolated in highly pure form. Each compound was dissolved in MeOH at a concentration of 200 ppm, and the LC-MS analysis conditions are listed in Table 1. LC-MS analysis is a powerful tool in metabolic profiling and metabolomics research and it can accurately determine the content of specific metabolites even at low levels in plant samples. LC-MS analysis was used previously to identify certain constituents of* A. tegmentosum*. The comparison of UV spectra, mass spectra, and retention times of the five types of compounds with the data of standard compounds led to their unambiguous assignments. Simultaneous analysis based on *R*_*t*_ of feniculin (**1**), avicularin (**2**), (+)-catechin (**3**), (−)-epicatechin (**4**), and 6′-*O*-galloyl salidroside (**5**) of 32.6, 34.0, 12.1, 17.7, and 21.3 min, respectively, was correlated with the molecular mass data and relative response. ESI-MS analysis of compounds feniculin (**1**), avicularin (**2**), (+)-catechin (**3**), (−)-epicatechin (**4**), and 6′-*O*-galloyl salidroside (**5**) showed intense [M+H]^+^ signals at 435.1, 435.1, 291.0, 291.0, and 453.1* m/z* values, respectively.

### 3.4. Anti-Inflammatory Activities Screening

#### 3.4.1. Effect of Five Compounds on RAW 264.7 Cell Viability

**Figure 8:**
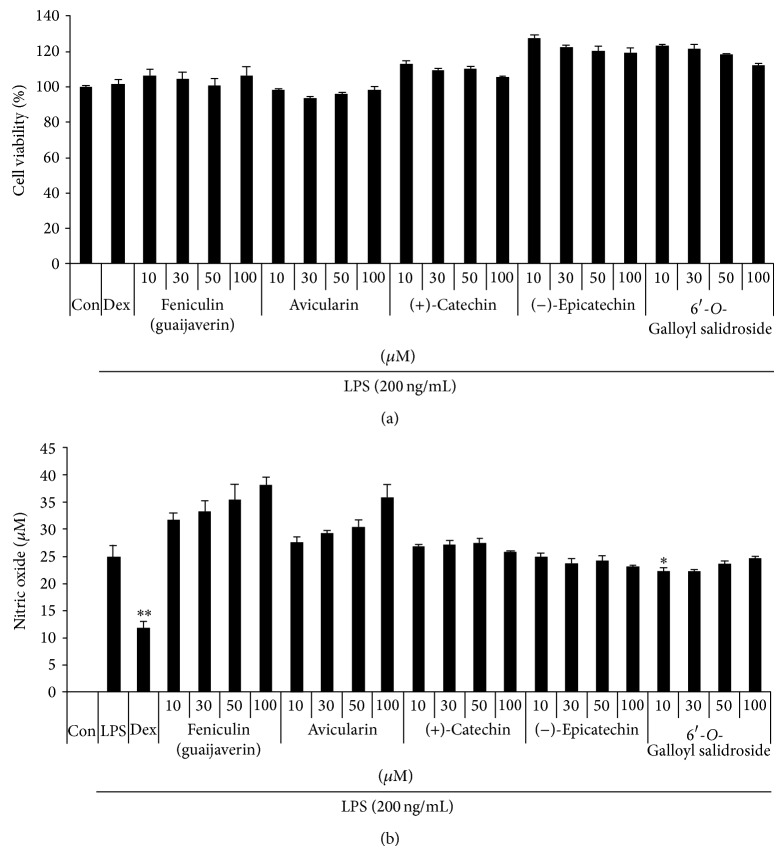
Effect of five compounds on (a) cell viability and LPS-induced (b) NO production in RAW 264.7 cells. RAW 264.7 cells were pretreated with five compounds for 30 min before incubation with LPS for 24 h. (a) Cytotoxicity was evaluated by an MTT assay. (b) The culture supernatant was analyzed for nitrite production. As a control, the cells were incubated with vehicle alone. Data shows mean ± SE values of triplicate determination from independent experiments. ^*^ *P* < 0.01 and ^**^ *P* < 0.001 were calculated from comparing with LPS-stimulation value.

We evaluated the cytotoxicity of five compounds using the MTT assay to determine the optimal concentration effective for anti-inflammation with minimum toxicity. As shown in Figure 8(a), all the five compounds did not affect cell viability up to 100 *μ*M, indicating that the compounds were not toxic to cells.

#### 3.4.2. Effect of Five Compounds on NO Production in LPS-Stimulated RAW 264.7 Macrophages

We evaluated the effects of five compounds on NO secretion in LPS-stimulated RAW 264.7 cells. The cells were pretreated with five compounds at various concentrations prior to the LPS stimulation, and NO production was measured. As a positive control, we employed 10 *μ*M dexamethasone, which is widely employed as an anti-inflammatory agent. All the compounds, except 6′-*O*-galloyl salidroside, did not show any inhibitory effect on LPS-induced NO production (Figure 8(b)).

#### 3.4.3. Effect of Five Compounds on LPS-Induced Inflammatory Cytokines Production

**Figure 9:**
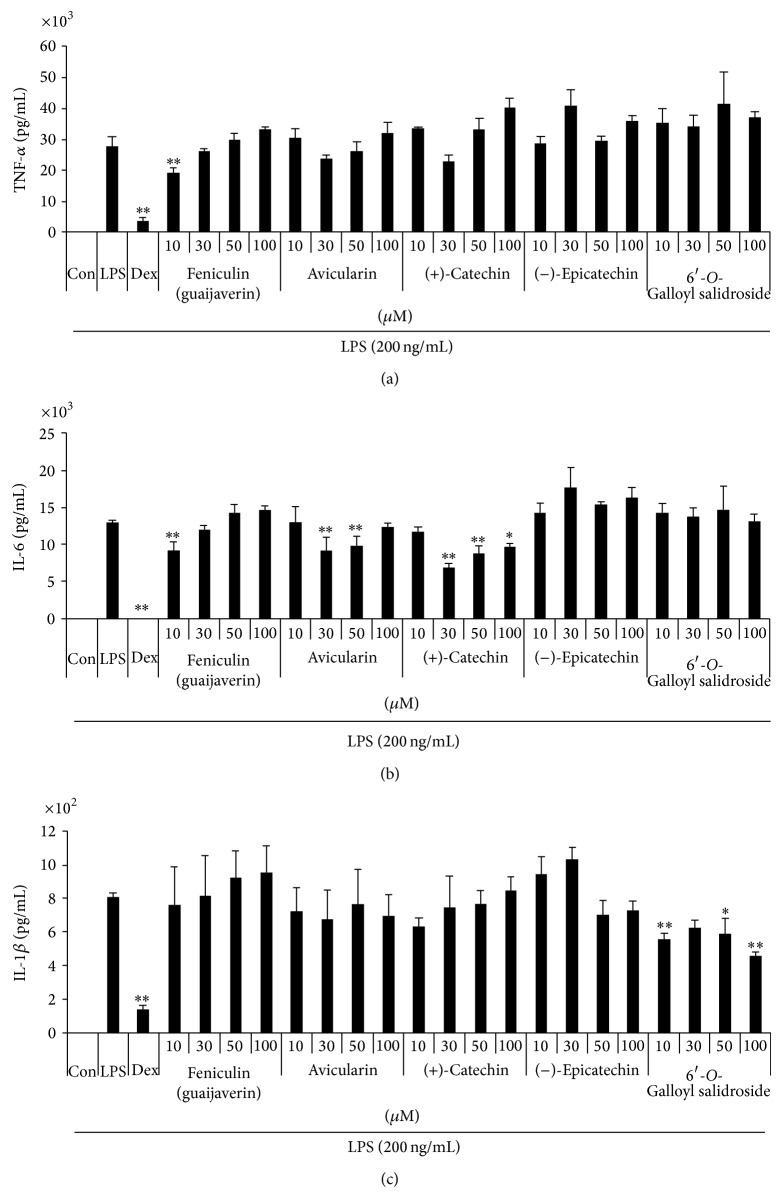
Effect of five compounds on the production of (a) TNF-*α*, (b) IL-6, and (c) IL-1*β* cytokines in macrophages. Cells were pretreated with five compounds for 30 min before being incubated with LPS for 24 h. Production of cytokines was measured by ELISA. Data shows mean ± SE values of duplicate determinations from three independent experiments. ^*^ *P* < 0.01 and ^**^ *P* < 0.001 were calculated from comparing with LPS-stimulation value.

The inhibitory effect of the five compounds on the production of inflammatory cytokines, another parameter of inflammation, was investigated. In this study, we examined the effect of the five compounds on TNF-*α*, IL-6, and IL-1*β* expressions.  Figure 9(a) shows that feniculin slightly repressed TNF-*α* production at a concentration of 10 *μ*M (Figure 9(a)). However, avicularin, (+)-catechin, (−)-epicatechin, and 6′-*O*-galloyl salidroside did not show any inhibitory effect on LPS-induced TNF-*α* production. As shown in Figure 9(b), consistent with TNF-*α* results, feniculin slightly repressed IL-6 production at a concentration of 10 *μ*M. Moreover, avicularin inhibited IL-6 production at concentrations of 30 and 50 *μ*M. (+)-Catechin inhibited IL-6 production at 30 *μ*M or more. (−)-Epicatechin and 6′-*O*-galloyl salidroside did not show any inhibitory effect on LPS-induced IL-6 secretion. However, all the compounds except 6′-*O*-galloyl salidroside did not inhibit IL-1*β* (Figure 9(c)).

## 4. Conclusions

This study showed that among the soluble fractions from the hot water extract of* A. tegmentosum*, the EA-soluble fraction possessed the highest bioactivity and free radical-scavenging activities. Compounds in the dried twigs of* A. tegmentosum *were extracted with hot water and partitioned successively using DCM, EA,* n*-BuOH, and water. The content of useful compounds in* A. tegmentosum* was remarkably higher in EA the extract (1.24 g). Five phenolic compounds were isolated by the silica gel, octadecyl silica gel, and Sephadex LH-20 column chromatography. The chemical structures of the isolated compounds were determined by spectroscopic methods, such as ^1^H-NMR, ^13^C-NMR, and LC/MS and were confirmed as feniculin (**1**), avicularin (**2**), (+)-catechin (**3**), (−)-epicatechin (**4**), and 6′-*O*-galloyl salidroside (**5**) by comparison of spectral data with those of references. The EA extract of* A. tegmentosum *containing five phenolic compounds exhibiting the best bioactivity was further monitored by an on-line screening HPLC-ABTS^+^assay method. Compounds** 1** and** 2** were isolated for the first time, and their anti-inflammatory activities were evaluated. Moreover, the on-line screening HPLC-ABTS^+^ assay method was rapid and efficient to search for bioactive compound from* A. tegmentosum. *Furthermore, (+)-catechin and 6′-*O*-galloyl salidroside exhibited the inhibitory activities on inflammatory mediator production such as TNF-*α*, IL-6, and IL-1*β* cytokines. In conclusion,* A. tegmentosum *can be used as a basic material for the development of new drugs in OMHs.

## Figures and Tables

**Table 1:** LC-MS operating condition.

Instrument	Condition
Column	HECpTOR-M-RS-tech C_18_ (100 × 4.6 mm, 5 *μ*m)
Oven temp. (°C)	40
Flow rate (mL/min)	0.5
Injection vol. (*μ*L)	10
Ionization source (positive mode)	API-ES
Fragmentor voltage (V)	70
Quadrupole temp. (°C)	99
Capillary voltage (V)	3000
Nebulizer pressure (psi)	35
Drying gas temp. (°C)	350
Mass rang (*m*/*z*) scan mode	200~500
Mobile phase (%)	A: 0.1% TFA in water, B: acetonitrile
Gradient elution composition (%)	(B): 10–25 (0–35 min), 25–10 (35–37 min), 10 (37–45 min)

**Table 2:** Analysis of gradient elution conditions with RP-HPLC.

Instrument	Condition	
Reverse phase column	RS-Tech Optimapak C_18_ (4.6 × 250 mm, 5 *µ*m)	
Oven temp. (°C)	40	
Flow rate (mL/min)	1.0	
Mobile phase (%)	A: 0.1% TFA in water, B: acetonitrile	
UV absorbance (nm)	210, 254, 280, 320	

Time (min)	Solvent composition (%)	
	0.1% TFA in water (A)	Acetonitrile (B)

0	90	10
50	60	40
60	60	40
70	90	10

**Table 3:** ^
13^C-NMR chemical shifts of compounds **1–5** isolated from *Acer  tegmentosum*.

Number of carbon	**1**	**2**	**3**	**4**	**5**
	CD_3_OD 100 MHz	CD_3_OD 150 MHz	CD_3_OD 150 MHz	CD_3_OD 150 MHz	CD_3_OD 150 MHz
1					130.56
2	158.70	159.44	82.81	79.86	130.83
3	135.65	135.00	69.78	67.47	116.08
4	179.48	180.09	28.47	29.25	156.52
5	163.05	163.19	157.54	157.66	116.08
6	99.88	99.95	96.28	96.38	130.83
7	166.07	166.25	157.78	157.98	36.33
8	94.70	94.84	95.50	95.88	72.20
9	158.43	158.69	156.88	157.35	
10	105.63	105.69	100.81	100.06	
1′	122.89	123.09	132.19	132.28	
2′	117.45	116.54	115.23	115.88	
3′	145.95	146.47	146.19	145.76	
4′	149.96	149.96	146.21	145.92	
5′	116.17	116.54	116.08	115.31	
6′	123.03	123.20	120.03	119.38	
Sugar-1′′	104.63	109.59			104.39
2′′	72.87	83.41			74.99
3′′	74.13	78.76			77.84
4′′	69.11	88.10			71.67
5′′	66.94	62.64			75.34
6′′					64.70
Galloyl-1′′′					121.37
2′′′					110.16
3′′′					146.44
4′′′					139.78
5′′′					146.44
6′′′					110.16
–COO–					168.31

**Table 4:** Extract amount and yield in ethyl acetate (EA) fraction sample.

Extraction solvent	Fraction (number)	Yield amount (mg)	Fraction (number)		Yield amount (mg)	Fraction (number)		Yield amount (mg)	Fraction (number)		Yield amount (mg)	Fraction (number)		Yield amount (mg)	Fraction (number)		Yield amount (mg)
EA phase	1	39.0	6	1	32.1	7	1	9.8	7-6	1	2.7	9	1	63.1	9-3	1	2.5
	2	101.8		2∗	6.0∗		2	164.0		2∗	30.7∗		2	22.8		2∗	60.3∗
	3	65.7		3	5.2		3	3.7		3	3.2		3	77.8		3	7.8
	4	23.5		4∗	5.9∗		4	6.2		4∗	14.2∗		4	8.6		4	12.3
	5	40.9		5	1.8		5	7.9		5	2.4		5	8.5			
	6	55.5		6	1.3		6	53.1		6	11.2		6	8.3			
	7	260.3		7	2.2		7	7.2					7	4.7			
	8	42.1					8	5.6					8	4.3			
	9	237.5					9	4.9					9	4.0			
	10	97.5					10	2.1					10	5.7			
	11	132.4											11	10.3			
	12	255.2											12	9.0			
													13	2.7			

^*^Shown for five kind compounds from major extract.

**Table 5:** Extracts and bioactivity efficiency by on-line screening HPLC-ABTS in positive and negative peak area.

Compounds name	Retention time (*R*_*t*_; min)	Positive peak area (mAU)	Negative peak area (mAU)	Figure number
Avicularin E-6-4	10.650	12.3413	5.7326	Figure 5 (EA phase; complex compounds)
Feniculin E-6-2	13.870	14.6317	5.2711	
(+)-Catechin E-7-6-2	15.880	20.5975	14.0358	
(−)-Epicatechin E-7-6-4	24.167	3.6339	0.4618	
6′-*O*-Galloyl salidroside E-9-3-2	24.983	2.8776	0.3851	

Avicularin E-6-4	10.700	3.6358	12.7703	Figure 6 (EA phase; isolation compounds)
Feniculin E-6-2	13.884	2.7657	10.5891	
(+)-Catechin E-7-6-2	15.928	7.3008	11.8252	
(−)-Epicatechin E-7-6-4	24.217	4.1437	3.0740	
6′-*O*-Galloyl salidroside E-9-3-2	25.062	5.1196	4.1546	
